# Validation of a Simulated Commercial Frying Process to Control *Salmonella* in Donuts

**DOI:** 10.1089/fpd.2018.2440

**Published:** 2018-12-11

**Authors:** Lakshmikantha H. Channaiah, Minto Michael, Jennifer Acuff, Keyla Lopez, Daniel Vega, George Milliken, Harshavardhan Thippareddi, Randall Phebus

**Affiliations:** ^1^ AIB International, Inc., Manhattan, Kansas.; ^2^ Food Science Institute, Kansas State University, Manhattan, Kansas.; ^3^ Department of Statistics, Kansas State University, Manhattan, Kansas.; ^4^ Poultry Science Department, University of Georgia, Athens, Georgia.

**Keywords:** *Salmonella*, donuts, flour, frying, validation

## Abstract

This study validated a typical commercial donut frying process as an effective kill-step against a 7-serovar *Salmonella* cocktail (Newport, Typhimurium, Senftenberg, Tennessee, and three dry food isolates) when contamination was introduced through inoculated flour. The bread and pastry flour mix (3:1) was inoculated with the *Salmonella* cocktail, and subsequently dried back to original preinoculation moisture content, achieving a *Salmonella* population of 7.6 log CFU/g. Inoculated flour was used to prepare a typical commercial donut batter, which was fried using 375°F (190.6°C) oil temperature. No viable *Salmonella* was detected using an enrichment plating protocol in the donuts after 2 min of frying, resulting in >7-log reduction in *Salmonella* population. The internal donut temperature increased from ∼30°C to ∼119°C at the end of 2 min of frying. The water activities of the donut crumb and crust after 2 min of frying, followed by 30 min of ambient air cooling, were 0.944 and 0.852, respectively. The donut pH after ambient-air cooling was 5.51. The D- and *z*-values of the *Salmonella* cocktail in donut dough were determined using thermal-death-time disks and temperature-controlled water baths. The D-values of the cocktail were 8.6, 2.9, and 2.1 min at 55°C, 58°C, and 61°C, respectively, whereas the *z*-value was 10°C. This study validated that >7-log reduction could be achieved if donuts are fried for at least 2 min in the oil at 190.6°C, and calculated D- and *z*-values present the heat resistance of *Salmonella* in donut dough at the start of the frying processes. However, results from this study should not be extrapolated when donut composition and frying parameters are changed significantly.

## Introduction

Contamination of raw ingredients used in processed foods can occur, and pathogens such as *Salmonella* and Shiga toxin-producing *Escherichia coli* can be introduced into bakery products through a wide range of ingredients such as egg, milk products, flour, chocolate, coconut, peanut butter, fruit, spices, and yeast (Ahmad *et al.*, 2000; Akins, 2014). Bakery products are not frequently linked to foodborne illness outbreaks; however, the potential presence of pathogens in various ingredients creates a public health risk if the product is improperly baked or fried. In addition, the ability of *Salmonella* to survive under various environmental stress conditions, such as low water activity (a_w_) and sublethal heat treatments, for lengthy periods of time makes it a challenging foodborne hazard for food manufacturers (Podolak *et al.*, 2010; Van Doren *et al.*, 2013). Although baking, cooking, roasting, frying, and boiling are generally considered effective kill-steps in controlling potential foodborne pathogens in food products, formal scientific evidence or validation of most of these processes for the inactivation of foodborne pathogens in bakery products has not been thoroughly investigated (Channaiah *et al.*, 2016). The U.S. Food and Drug Administration's Food Safety Modernization Act (FSMA) shifted the main focus from reacting to food safety failures to proactively preventing these failures. According to the FSMA, food processors must validate all preventive controls that are process steps and critical for food safety based on the scientific evidences (FDA, 2015). Food manufacturers in the United States or those exporting to the United States need such validation documentation to support their food safety programs under the requirements of FSMA (FDA, 2015).

The main purpose of this study was to validate a frying process simulating commercial donut manufacturing to control *Salmonella* contamination potentially introduced through flour; thus, to produce microbiologically safe ready-to-eat donuts. In this study, a yeast-raised donut recipe was used because it is one of the most popular bakery products consumed in the United States and Canada. Through a literature search, no other study was found validating a bakery product with frying as a kill-step for *Salmonella*. The specific research objectives of this study were to (1) validate the frying process as a kill-step to control *Salmonella* in donuts and (2) determine the heat resistance parameters (D-values and *z*-values) of *Salmonella* in proofed donut dough. In addition, the a_w_, pH, and proximate composition of donuts were also determined.

## Materials and Methods

### Experimental and statistical designs

This research was divided into the following three studies: (1) validation of frying as a kill-step to inactivate a 7-serovar *Salmonella* cocktail in donuts during frying using 375°F (190.6°C) oil temperature for 2 min; (2) determination of the internal temperature profile, pH and a_w_ of donuts during frying; and (3) determination of D- and *z*-values of the 7-serovar *Salmonella* cocktail in proofed donut dough.

For the frying validation study, donut dough was prepared using an inoculated (*Salmonella* cocktail) bread and pastry flour mix. The donuts were fried in soybean oil at 190.6°C for 2 min followed by 30 min of ambient air cooling (F+C), with product sampling at 1 and 2 min of frying and at F + C to enumerate the surviving *Salmonella* population. This study utilized a randomized complete block experimental design with three replications as blocks and six sampling times as treatments (inoculated flour; pre- and postproof dough; 1 and 2 min of frying; and F+C). For the temperature profiling, pH and a_w_ determination study, noninoculated flour mix was used to prepare donut dough. This study was also designed as randomized complete block (three replications) with four sampling times as treatments (pre- and postproof dough; 2 min of frying; and F+C). Microbial, pH and a_w_ data were statistically analyzed by analysis of variance at *p* ≤ 0.05 using SAS version 9.3 (SAS Institute, Cary, NC). An additional donut frying study was conducted using noninoculated flour mix to provide samples after frying and F + C for proximate analyses.

The mean D- and *z*-values were calculated from the linear regression graphs plotted using Microsoft Excel 2011 (Microsoft Corp., Redmond, WA) for each replication separately. This study utilized a randomized complete block design with three replications and microbial plating was done in duplicate.

### *Salmonella* cultures and flour inoculation

Three serovars of *Salmonella* were obtained from the American Type Culture Collection (ATCC; Newport 6962, Senftenberg 775 W 43845 and Typhimurium 14028), and *Salmonella* Tennessee and three dry pet food isolates were obtained from Richter International, Inc. (Columbus, OH). These *Salmonella* serovars were selected because of their relatively high heat resistance, association with foodborne illness outbreaks, and/or isolation from low moisture food environment. The working cultures were propagated individually following the method described by Channaiah *et al.* (2017).

The bread (300 g) and pastry (100 g) flours were weighed into a sanitized sealable plastic tub (9.4 L, Rubbermaid, Atlanta, GA), mixed, and spread into a uniform layer. Flour mix was then mist-inoculated inside the biosafety cabinet as described by Channaiah *et al.* (2017).

### Donut dough preparation

All ingredients and the dough formula used for the donut preparation were provided by AIB International, Inc., Manhattan, KS (Table 1). Flour mix and other dry ingredients were weighed into a sanitized mixing bowl (Artisan^®^, KitchenAid^®^, St. Joseph, MI) and mixed with a sanitized spatula. Yeast, shortening, and water were then added to the dry ingredients, the mixing bowl and paddle were attached to the mixer, and the ingredients were mixed for 1 min at speed-1, followed by 5 min of mixing at speed-2. The dough was rounded into a ball, placed into a greased bowl, covered with aluminum foil, and rested at room temperature (∼25°C) for 45 min. The dough was then rolled into a ½-inch (12.7 mm) sheet, and rested for 10 min at room temperature. The dough was resheeted, cut into shape using a donut cutter [3 inch (76.2 mm) diameter with 1 inch (25.4 mm) inner hole diameter], placed onto a frying screen (four donuts per frying batch), and placed inside a proofing cabinet preset at 100°F (37.8°C) and 60–70% relative humidity for 30 min.

**Table 1.** Ingredients and Formula Used for Donut Dough Preparation

*Ingredient*	*Grams*
Bread flour	150
Pastry flour	50
Granulated sugar	24
Nonfat dry milk	8
Salt	4
Soy flour, defatted	6
Potato flour	10
Egg yolk solids	6
Sodium stearoyl lactylate	1
Yeast, fresh compressed	12
All purpose shortening	16
Water	116

### Donut frying and temperature monitoring

The donut frying parameters (oil temperature and frying time) were determined after a series of frying trials mimicking the end-product quality of commercially prepared donuts. The oil and internal donut temperatures during frying were monitored and recorded using fine-gauge thermocouples [Type-T Thermocouples (Omega Engineering, Inc., Stamford, CT)] connected to an eight-channel data logging system (USB-TC with MCC DAQ software, Measurement Computing, Norton, MA). During temperature monitoring, the data logger recorded temperatures every second. For monitoring internal donut temperatures during frying and ambient-air cooling, the thermocouples were inserted from the side into the center of two randomly selected donuts. The proofed donuts on the frying screen with attached thermocouples were transferred into the hot soybean oil at 375°F (190.6°C) and fried for 1 min on each side, followed by 30 min of ambient air-cooling on a sanitized wire rack.

### Frying validation

For frying validation, donuts (prepared from inoculated flour mix) were randomly sampled at 1 and 2 min of frying, and at F+C. At each sampling point, donuts were quickly removed from the hot oil and transferred into stomacher bags containing 100 mL of chilled (∼4°C) 0.1% peptone solution, hand massaged for 1 min to arrest additional thermal microbial destruction, and analyzed to determine the surviving *Salmonella* population.

### pH, a_w_, and proximate analyses of donuts

For pH and a_w_ measurements, samples were taken at pre- and postproofing, after 2 min of frying, and F+C. Donut samples after 2 min of frying and F + C were immediately separated into crust (external) and crumb (internal) components, and analyzed. The sample pH and a_w_ were measured as described by Channaiah *et al.* (2017). An additional donut frying was conducted, sampled at the previously stated times, and sent to the analytical laboratory in the Animal Sciences and Industry Department at Kansas State University for the proximate analyses of moisture, fat, protein, and starch.

### D- and z-values determination

D- and *z*- values of the 7-serovar *Salmonella* cocktail in postproof donut dough were determined following the method described by Channaiah *et al.* (2017) using ∼6 g samples in thermal-death-time (TDT) disks and temperature-controlled water baths.

### *Salmonella* enumeration

For the frying validation study, *Salmonella* population was enumerated on injury-recovery [BHI agar overlaid with Xylose lysine deoxycholate (XLD) agar] and selective media [XLD agar], whereas for the D- and *z*-values study, only injury-recovery media was used to enumerate *Salmonella* populations. The *Salmonella* enumeration and enrichment were conducted according to Channaiah *et al.*, 2017.

## Results and Discussion

Although, frying is an ancient and popular food preparation process, only a handful of researchers have explored the science behind validating a frying process in French fries (Palazoğlu and Gökmen, 2008), breaded pork patties (Osaili *et al.*, 2007), meatballs (Porto-Fett *et al.*, 2016), and in tortilla chips (Chen and Moreira, 1997) for various end-use quality parameters. In general, a better understanding of the product internal temperature and frying time are important in improving the quality as well as food safety parameter of the final product (Chen and Moreira, 1997; Osaili *et al.*, 2006; Wang *et al.*, 2015; Porto-Fett *et al.*, 2016). Most of the published frying validation studies focused mainly on the change in physical and chemical parameters of the fried food product and the oil (Chen and Moreira, 1997; Osaili *et al.*, 2007; Sébédio and Juaneda, 2007). However, considerably fewer studies have focused on validating a frying process as an effective kill-step for pathogen control (Osaili *et al.*, 2006; Porto-Fett *et al.*, 2016). To the best of our knowledge, this is the first frying validation study involving a bakery product for *Salmonella* destruction.

### Donut frying temperature profile

**FIG. 1.**
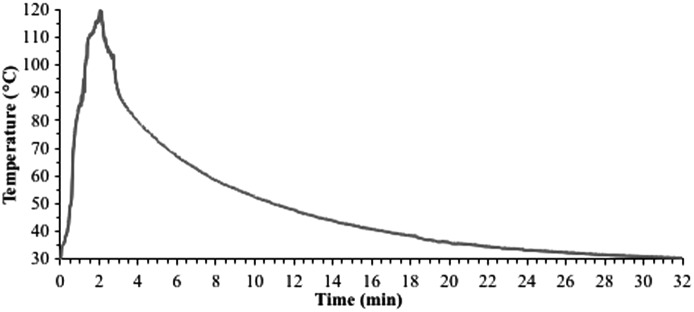
Temperature profile of donuts (internal) during 2 min of frying at 375°F (190.6°C) oil temperature followed by 30 min of ambient air-cooling.

The soybean oil temperature before the start of the frying process was maintained at ∼190°C for ∼30 min. After introducing donuts into the oil, the oil temperature decreased to ∼178°C at the end of 2 min of donut frying. The mean donut internal temperature at the start of frying was ∼30°C and increased to ∼119°C after 2 min of frying (Fig. 1). After 30 min of ambient-air cooling, the donut temperature decreased back to ∼30°C.

### Donut pH, a_w_, and proximate analyses

**FIG. 2.**
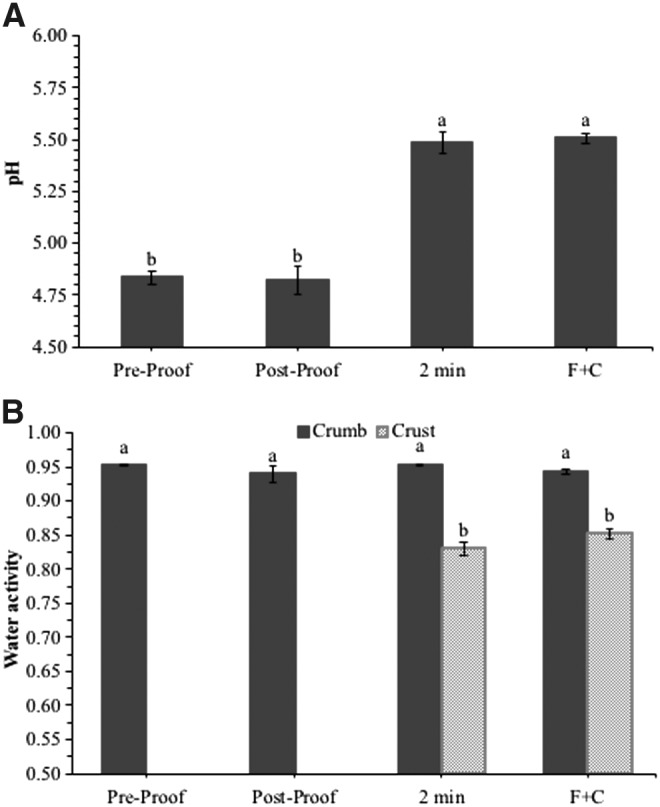
pH and water activity (mean ± SE) of pre- and postproof donut dough and donuts after 2 min of frying at 375°F (190.6°C) oil temperature followed by 30 min of ambient air-cooling (F+C). **(A, B)** Bars with different letters are statistically different (*p* ≤ 0.05).

The pH and a_w_ of pre- and postproof donut dough, and donuts after 2 min of frying and F + C are presented in Figure 2. The pH of postproof dough (4.82 ± 0.07) was similar to that of preproof dough (4.83 ± 0.04). However, the donut pH increased to 5.48 ± 0.05 and 5.51 ± 0.03 at the end of 2 min of frying and F+C, respectively. This pH increase in the fried donuts could be attributed to the oil absorbed by the donuts during the frying process. The pre- and postproof donuts had similar a_w_ (0.953 ± 0.001 and 0.940 ± 0.013, respectively). The a_w_ of the donut crumb after frying and F + C (0.953 ± 0.001 and 0.944 ± 0.004, respectively) remained similar to that of the dough; whereas, the a_w_ of the donut crust decreased to 0.830 ± 0.009 and 0.852 ± 0.007 after frying and F+C, respectively.

The proximate analyses of donut dough and fried donuts are presented in Table 2. As expected, at F + C the moisture content of donut was lower than that of dough with greater moisture in the crumb compared to the crust. As the frying oil absorbed into the donut during the frying process, the fat content of the donut at F + C was greater than that of dough, with greater fat in the donut crust.

**Table 2.** Proximate Analyses of Noninoculated Pre- and Postproof Donut Dough and Donuts After 2 Min of Frying at 375°F (190.6°C) Oil Temperature, Followed by 30 Min of Ambient Air-Cooling (F+C)

*Sample*	*% Moisture*	*% Fat*	*% Protein*	*% Starch*
Dough				
Preproof	41.3	5.9	9.1	39.0
Postproof	41.0	4.8	9.9	40.5
2 min				
Crumb	33.4	19.7	7.7	32.2
Crust	13.6	40.3	6.9	29.9
F + C				
Crumb	32.1	17.5	7.9	30.8
Crust	18.5	31.6	8.2	32.9

### Frying validation

**FIG. 3.**
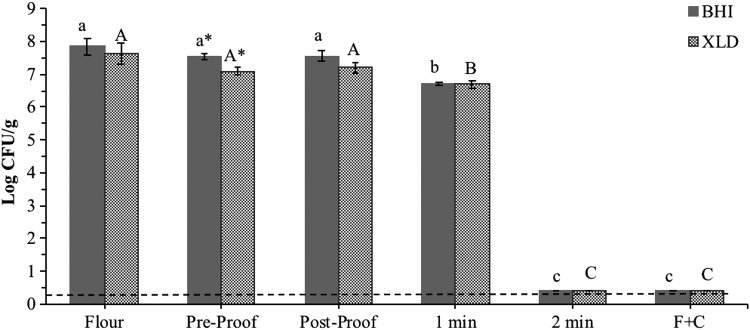
7-serovar *Salmonella* cocktail population in inoculated flour, dough, and donuts during 2 min of frying at 375°F (190.6°C) oil temperature, followed by 30 min of ambient air-cooling (F+C). **(A–C)** Values with different letters are significantly different (*p* ≤ 0.05) within the respective medium. *Values within a respective sampling point are different (*p* ≤ 0.05).—–: 0.40 log CFU/g detection limit. No viable *Salmonella* was detected (by enrichment plating) from donuts after 2 min of frying.

The 7-serovar *Salmonella* cocktail contained 11.1 ± 0.10 log CFU/mL, and inoculated flour contained 7.6 ± 0.32 log CFU/g (as enumerated on injury-recovery media). As flour was inoculated at such high levels, the presence of *Salmonella* in other ingredients was not tested because *Salmonella* population enumerated in the current study would be directly due to the artificial inoculation. The *Salmonella* populations in the postproof dough (7.6 ± 0.15 and 7.2 ± 0.16 log CFU/g as determined on injury-recovery and selective media, respectively) were similar to that in the inoculated flour (Fig. 3). After 1 min of frying, the *Salmonella* population in donuts decreased to 6.7 log CFU/g as enumerated on both injury-recovery and selective media (Fig. 3). However, no viable *Salmonella* was detected in the donuts after 2 min of frying and F + C after enrichment, indicating the complete elimination of *Salmonella*. These results indicate that >7-log reduction of *Salmonella* was achieved in the donuts during the 2-min frying process. In a different study, Porto-Fett *et al.* (2016) evaluated the effect of deep-frying on inactivation of Shiga toxin-producing *Escherichia coli* (STEC) in meatballs. In this study, deep-frying fresh meatballs (40 g each) in canola oil at 176.7°C for 5.5 min resulted in 5-log reduction, and cooking of fresh meatballs at 176.7°C for 12.5 min also resulted in 5-log reduction.

FSMA regulations place a greater importance on the risk assessment and prevention of food safety failures, which requires food processors to validate the food safety critical processing steps. Although in-plant validations (using nonpathogenic surrogate microbial cultures) may be the most effective form of validation, food processors are often reluctant to introduce any kind of microbial cultures into their processing facilities, and selection of an appropriate surrogate is often a challenge. Therefore, the food industry tends to rely upon published laboratory- and/or pilot-scale studies that utilize pathogens of interest and/or their surrogates. The current study, along with similar validation studies conducted for various bakery products, can be used by the bakery industry to assess the pathogen control capabilities of their manufacturing processes. In similar validation studies, Channaiah *et al.* (2016, 2017) validated commercially simulated baking processes as kill-steps against *Salmonella* in hamburger buns and plain muffins when contamination was introduced via contaminated flour.

### D- and z-values

**FIG. 4.**
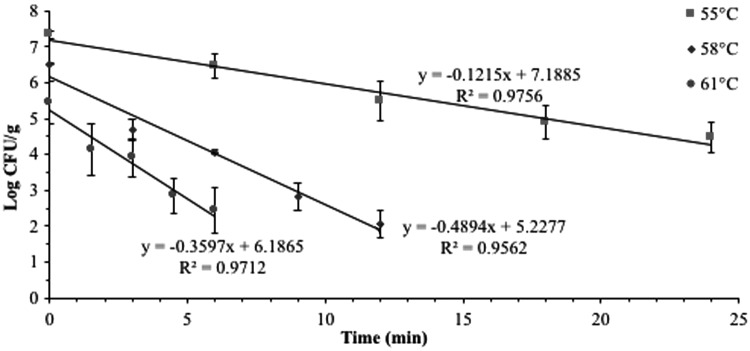
7-serovar *Salmonella* cocktail counts in proofed donut dough versus heating time as plated on injury-recovery media.

**FIG. 5.**
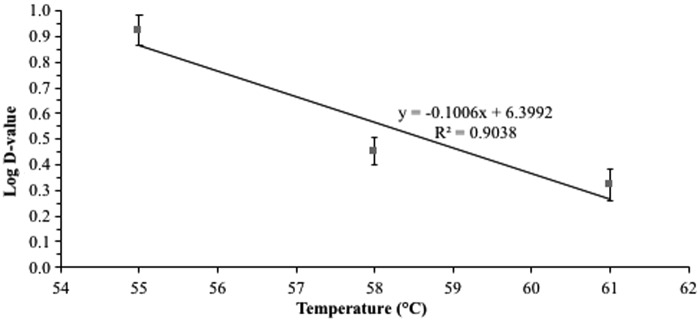
Log D-values of 7-serovar *Salmonella* cocktail in proofed donut dough versus dough temperature as calculated from counts on injury-recovery media.

The linear regression graphs used to calculate D- and *z*-values are presented in Figures 4 and 5; whereas, the calculated D- and *z*-values of 7-serovar *Salmonella* cocktail in proofed donut dough are presented in Table 3. The *R*^2^ values for all regression lines were >0.9. The D-values of *Salmonella* cocktail were determined at much lower temperatures (55–61°C) compared with the maximum internal donut temperature (∼119°C) reached during frying because during preliminary D-values studies, the time required to achieve high target temperatures resulted in significantly lower *Salmonella* populations that were inadequate to calculate D-values. Although extrapolating *Salmonella* thermal destruction at 119°C from the data determined at 55–61°C could cause some linearity problems, these D-values were determined to estimate the *Salmonella* heat resistance in donut dough at the start of the frying process. The D-values of *Salmonella* cocktail in dough were 8.6, 2.9, and 2.1 min at 55°C, 58°C, and 61°C, respectively, with a *z*-value of 10°C. In similar studies, Channaiah *et al.* (2016, 2017) determined that a 3-serovar *Salmonella* cocktail (Typhimurium, Newport, and Senftenberg) in proofed hamburger bun dough and plain muffin batter had D-values of 28.6 and 62.2, 7.6, and 40.1, and 3.1 and 16.5 min at 55°C, 58°C, and 61°C, respectively, and *z*-values of 6.6°C and 10.4°C, respectively. The differences in the D- and *z*-values in the current study (proofed donut dough) and those generated by Channaiah *et al.* (2016, 2017) for proofed bun dough and muffin batter could be attributed to differences in the *Salmonella* cocktails used in these studies. Moreover, the proximate compositions of hamburger bun dough (46.9% moisture, 8.5% protein, 3.8% fat, and 40.8% starch) and muffin batter (31.2% moisture, 6.3% protein, 8.9% fat, and 24.3% starch) in Channaiah *et al.* (2016, 2017) studies were different than that of donut dough in the current study. The lower D-values of *Salmonella* cocktail in proofed donut dough compared to that in muffin batter (Channaiah *et al.*, 2017) could also be attributed to the lower pH of proofed donut dough (4.82) than muffin batter (6.61) that would have facilitated thermal inactivation of *Salmonella* in donut dough. Channaiah *et al.* (2016) also demonstrated that the D-values of *Enterococcus faecium* in hamburger bun dough at 55°C, 58°C, and 61°C were approximately four to seven times greater than the 3-serovar *Salmonella* cocktail, and hence *E. faecium* can be used as a conservative surrogate strain for *Salmonella* to validate various thermal processes for bakery products if in-plant studies are desired.

**Table 3.** D-Values (min) and *z*-Value (°C) (Mean ± SE) of a 7-Serovar *Salmonella* Cocktail in Proofed Donut Dough as Calculated from the Counts Obtained by Plating on Injury-Recovery Media

*Item*	*Calculated value*
55°C	8.6 ± 1.20
58°C	2.9 ± 0.36
61°C	2.1 ± 0.28
z-value	10.0 ± 0.68

An earlier study (Osaili *et al.*, 2006) investigated thermal inactivation kinetics of *E. coli* O157:H7*, Salmonella* and *Listeria monocytogenes* in ready-to-eat chicken-fried beef patties. Osaili *et al.* (2006) reported an average D-values of 27.62 to 0.04 min for *E. coli* O157:H7, 67.68 to 0.22 min for *Salmonella*, and 81.37 to 0.31 min for *L. monocytogenes* at temperatures 55–70°C. The lower D-values for *Salmonella* cocktail reported in our study can be attributed to differences in food matrices used and fat content in chicken-fried beef patties. In addition to differences in food matrices and intrinsic factors, variation in the thermal resistance of the bacterial strains used to prepare the inoculum and experimental methodologies could account for differences in D-values.

## Conclusions

The current study demonstrated that frying donuts at >190°C oil temperature for at least 2 min will result in >7-log reduction in *Salmonella* population. This study can be used by commercial donut manufacturers utilizing similar operational parameters to fulfill the FSMA requirements to scientifically validate frying a critical processing step in donut manufacturing. The D- and *z*-values determined in this study can also give a clear understanding of *Salmonella* heat resistance in proofed donut dough, and can be used for optimizing donut frying processes. It should be noted that individual donut frying processes should be validated when donut proximate composition and frying parameters are different than those studied in the current research.
